# Efficacy and Safety of Upadacitinib in Moderate-to-Severe Atopic Dermatitis: A Meta-Analysis

**DOI:** 10.7759/cureus.64488

**Published:** 2024-07-13

**Authors:** Bikash R Meher, Archana Mishra, Biswanath Behera, Subashri Ponnusamy

**Affiliations:** 1 Pharmacology, All India Institute of Medical Sciences, Bhubaneswar, IND; 2 Dermatology, All India Institute of Medical Sciences, Bhubaneswar, IND

**Keywords:** upadacitinib, immune related skin disorder, jak inhibitor, systematic review and meta analysis, meta-analysis, atopic eczema, atopic dermatitis

## Abstract

Atopic dermatitis (AD) is a chronic, relapsing inflammatory skin disorder. Topical corticosteroids are the cornerstone of therapy in mild AD, whereas the JAK inhibitor upadacitinib is approved in the United States, Europe, and other countries for treating moderate-severe AD in adults and children over 12 years old whose disease is not adequately controlled with other systemic drugs, including biologics. The objective of this meta-analysis was to assess the overall efficacy and safety of upadacitinib in moderate to severe AD. All randomized controlled trials (RCTs) evaluating the efficacy and safety of upadacitinib in moderate to severe AD were included in the meta-analysis. The pooled analysis revealed a significant proportion of patients achieving Eczema Area and Severity Index-75 (EASI 75) (R.R. = 3.86; 95% CI = 3.12 to 4.78, p < 0.00001), EASI 100 (R.R. = 13.09; 95% CI = 7.40 to 23.17, p < 0.00001), Worst Pruritus Numerical Rating Score (WP-NRS) response (R.R. = 4.44; 95% CI = 3.72 to 5.29, p< 0.00001), and validated Investigator’s Global Assessment (v-IGA) (RR = 5.96; 95% CI = 4.79 to 7.41, p < 0. 00001) in the upadacitinib arm compared to the placebo arm. Moreover, the pooled analysis also suggested that treatment-emergent adverse events (TAEs) were relatively higher with upadacitinib than with placebo, but were mild and easily manageable (R.R. = 1.15; 95% CI = 1.09 to 1.23, p<0.00001). This meta-analysis showed that upadacitinib had a significant beneficial effect and tolerable adverse effect profile in patients with moderate and severe AD. Dose regimens of 15 mg and 30 mg seemed to have similar benefits. However, further trials are needed to assess long-term efficacy and safety profile.

## Introduction and background

Atopic dermatitis (AD) or atopic eczema is a chronic, recurrent inflammatory cutaneous disorder characterized by dry skin, intense itching, and eczematous lesions [1]. AD compromises the quality of life (QoL) of affected patients and families and escalates overall health expenses. The incidence of AD is rising globally and has increased two to three-fold in developed nations in the last few decades. It is more common in children, affecting about 20% and 10% of children and young adults, respectively, across the world [2,3]. Abnormalities in the skin barrier, immunological dysregulation, and genetic and extrinsic triggers seem to play a pivotal role in pathogenesis [4,5]. The Janus kinase (JAK) signal transducers and activators of the transcription (STAT) pathway play a key role in the development of AD [6].

The management of AD involves both non-pharmacologic (patient education, wet wrap therapy, etc.) and pharmacologic intervention. Topical corticosteroids, along with calcineurin inhibitors like tacrolimus and pimecrolimus, are usually the first line of drug therapy [7,8]. In recent times, many novel agents have emerged as effective alternatives for the treatment of AD not responding to standard therapy. Dupilumab was the first approved biologic for the treatment of moderate-to-severe AD [9]. Of late, small molecules targeting the JAK pathway have also shown tremendous promise in the management of AD and received approval from regulatory agencies for the use of the same [10]. Upadacitinib is a second-generation JAK inhibitor preferentially selective for JAK1 than for JAK2 and JAK3, which received a nod from the U.S. Food and Drug Administration (US FDA) in 2022 for the treatment of moderate to severe AD patients aged 12 years and above.

Though few clinical trials have proven that upadacitinib is efficacious and safe in moderate to severe atopic dermatitis in adolescent and adult age groups, there has been a variation in the findings in those studies [11-14]. Given that, we conducted this meta-analysis to aggregate the overall result and assess the robustness of results from these trials.

## Review

Materials and methods

Protocol Development and Registration

This study was conceptualized based on Preferred Reporting Items for Systematic Review and Meta-Analysis Protocols (PRISMA-P) 2015 guidelines and performed according to the recommendation of the ‘‘Cochrane Handbook for the Systematic Review of Intervention Guidelines’’ [15]. Before the commencement of the study, the protocol was registered on PROSPERO (CRD42022372028).

Literature Search

Four reviewers independently carried out a detailed search to identify all studies reporting the efficacy and safety of upadacitinib treatment in AD. PubMed/MEDLINE, Web of Science, and Cochrane Library were searched for articles published up to December 2023 using a combination of keywords and Boolean operators like "Upadacitinib"[All Fields] AND ("Atopic eczema"[All Fields] OR "Atopic dermatitis"[All Fields]). We followed the standard Preferred Reporting Items for Systematic Reviews and Meta-Analysis (PRISMA) statement and Cochrane guidelines for the search process.

Study Selection

Studies: Randomized clinical trials (RCTs) comparing the effect of upadacitinib with placebo in patients with moderate to severe AD were included in this meta-analysis. Non-human studies, narrative reviews, case studies, commentaries, conference abstracts, and retrospective comparisons were not considered for this study. There was no study restriction based on ethnicity.

Participants: Patients of age 12 years or more of either gender with a diagnosis of moderate to severe AD and not responding adequately to topical medications were considered for this study.

Intervention

Upadacitinib 15 mg or 30 mg per day orally with or without topical corticosteroid therapy was considered an intervention for this meta-analysis.

Comparator

The group that received a placebo was used for comparison in this meta-analysis.

Outcomes

The primary outcome was an Eczema Area and Severity Index-75 (EASI-75) response at week 16 and the secondary outcomes were an EASI-100 response, validate-Investigator Global Assessment for Atopic Dermatitis (vIGA-AD) response, Worst Pruritus Numerical Rating Score (WP-NRS) response, and treatment-emergent adverse effects (TEAEs) at week 16.

*Study Selection and Data Collection* 

All collected publications were scrutinized by four authors (BRM, SM, AM, BB) separately for the titles, abstracts, and keywords. The chosen publications were evaluated for their eligibility and selected for data extraction. Any disagreement between the authors was sorted out through deliberation.

Data Extraction and Management

For this meta-analysis, three authors independently extracted the data and assessed the quality based on the guidelines of Cochrane Collaboration. If there was a dispute, it was resolved by discussing it with another author (BRM). Data extraction was executed using a specially designed format, which included reference, clinical trial identifier, study design, study period, number of participants, duration of treatment, patient characteristics, intervention and comparator, and outcome measures.

Data Analysis

Cochrane Review Manager, Revman (Version 5.4)’ was used for this meta-analysis [16]. The risk ratio (R.R.) of dichotomous outcomes was measured and expressed with their 95% confidence intervals. A random-effect meta-analysis model was employed to pool the estimate. The statistical heterogeneity of the enrolled studies was calculated using the I2 statistic. A P value of less than 0.05 was considered statistically significant.

Assessment of Risk of Bias of Included Studies

Four review authors independently evaluated the internal validity and methodological quality of eligible studies in accordance with the Cochrane risk-of-bias tool for randomized trials (RoB-2) tool, consensus was reached by resolving any differences by discussion. The overall risk of bias was considered as high, low, or unclear based on the cumulative assessment of individual domains.

Assessment of Publication Bias

We used Egger's test to quantify the degree of potential publication bias across the included studies.

Summary of Findings

The grade of evidence was established using the GRADE pro G.D.T tool following five grade considerations ‘‘risk of bias, inconsistency, imprecision, indirectness, and publication bias’’ in the methodology and result of the selected studies [17].

Results

Search Results

**Figure 1:**
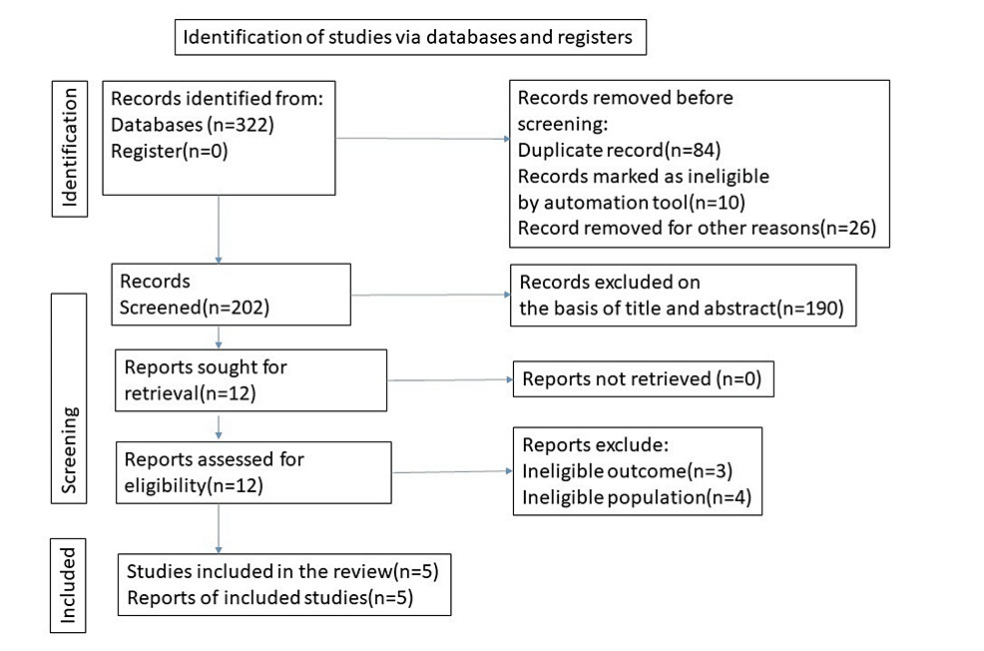
PRISMA flow diagram of the study selection process PRISMA: Preferred Reporting Items for Systematic Reviews and Meta-Analyses

An initial database search with the above-mentioned keywords identified a total of 322 publications. After the exclusion of duplicates, 202 records were screened by titles and abstracts, and 12 full articles were assessed for eligibility. We excluded seven studies, as these did not meet the inclusion criteria of our study. Hence, five studies were selected for this meta-analysis. The search methodology was documented in the PRISMA flow chart as illustrated in Figure 1.

Description of Studies

We considered five studies with a total number of 3023 moderate to severe AD patients for this meta-analysis. All the studies were published in the year 2021 except one study (Guttman-Yassky et al. (2020)), and all compared the clinical outcomes of upadacitinib with that of a placebo. Out of five enrolled studies, four were phase 3, three-arm, randomized, placebo-controlled trials with upadacitinib 30 mg and 15 mg or placebo, whereas Guttman-Yassky et al.'s study (2020) was a phase 2b study consisting of four arms (upadacitinib 30 mg, 15 mg, 7.5 mg, or placebo). The article by Guttman-Yassky et al. (2021) consisted of two separate studies, named Measure Up 1 and Measure Up 2, and those by Reich et al. and Katoh et al. were known as AD Up and Rising Up, respectively. All five included studies had enrolled participants 12 years or older with treatment for 16 weeks. Data were extracted from the selected studies and analyzed for the 15 mg and 30 mg groups of upadacitinib and placebo (Table 1).

**Table 1:** Characteristics of studies included in the meta-analysis ADerm-SS, atopic dermatitis symptom scale; CDLQI, children’s dermatology life quality index; DLQI, dermatology life quality index; EASI, eczema area and severity index; HADS, hospital anxiety and depression scale; vIGA, validated investigator global assessment; WPNRS, worst pruritus numerical rating scale; POEM, patient-oriented eczema measure; PSAAD, pruritus and symptoms assessment for atopic dermatitis; RCT, randomized controlled trial; SCORAD, scoring atopic dermatitis; TEAE, treatment-emergent adverse event

Authors	Clinical trial identifier	Phase	No. of participants	Intervention	Duration of treatment	Efficacy outcome	Safety outcome
Guttman- Yassky et al. (2020) [11]	NCT02925117	Phase 2b	167	Treatment: oral upadacitinib, 7.5 mg,15 mg,30 mg once daily Placebo: oral control vehicle once daily	16 weeks	EASI, vIGA-AD WPNRS, SCORAD, POEM, DLQI	TAEs
Guttman- Yassky et al.-1 (2021) [13]	NCT03569293 (Measure Up 1)	Phase 3	847	Treatment: oral upadacitinib, 15 mg, 30 mg once daily Placebo: oral control vehicle once daily	16 weeks	EASI, vIGA-AD WPNRS, SCORAD, ADerm-SS TSS-7 POEM	TAEs
Guttman- Yassky et al.-2 (2021) [13]	NCT03607422 (Measure Up 2)	Phase 3	836	Treatment: oral upadacitinib, 15 mg, 30 mg once daily Placebo: oral control vehicle once daily	16 weeks	IGA, EASI, WNRS, PSAAD, DLQI, CDLQI, POEM, and HADS	TAEs
Reich et al. 2021 [12]	NCT03568318 (AD Up)	Phase 3	901	Treatment: oral upadacitinib, 15 mg, 30 mg once daily Placebo: oral control vehicle once daily	16 weeks	EASI, vIGA-AD WPNRS	TAEs
Katoh et al 2021 [14]	(Rising Up)	Phase 3	272	Treatment: oral upadacitinib, 15 mg, 30 mg once daily Placebo: oral control vehicle once daily	16 weeks	EASI, vIGA-AD WPNRS	TAEs

Risk of Bias in the Included Studies

All the included studies exhibited a "low" risk of bias for all the domains. Therefore, in overall judgment, the enrolled studies were considered to have a low risk of bias (Table 2).

**Table 2:** Risk-of-bias assessment RoB-2: version 2 of the Cochrane risk-of-bias tool for randomized trials (RoB 2)

RoB-2	RCTs	D1	D2	D3	D4	D5	Overall
	Guttman- Yassky et al. (2020) [11]	L	L	L	l	L	L
	Guttman-Yassky et al.-1 (2021) [13]	L	L	L	L	L	L
	Guttman-Yassky et al.-2 (2021) [13]	L	L	L	L	L	L
	Reich et al. 2021 [12]	L	L	L	L	L	L
	Katoh et al. 2021 [14]	L	L	L	L	L	L
	Domains: D1: Bias due to randomization process, D2: Bias due to deviation, D3: Bias due to missing outcome, D4: Bias due to measurement of outcome, D5: Bias due to selection of reported result			Low (L): Some concern, (SC): High (H):			

Efficacy Outcomes

**Figure 2:**
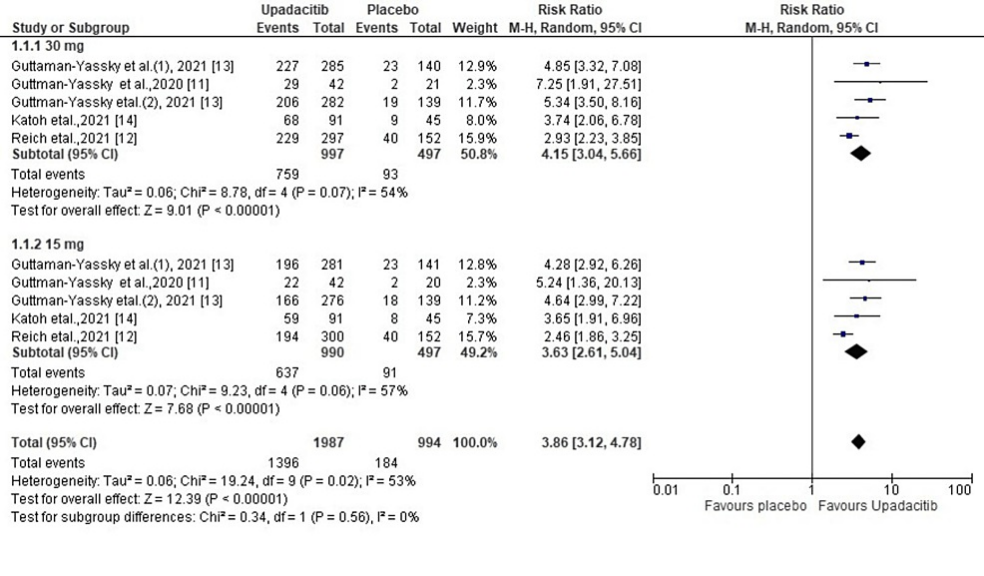
Forest plot of the included studies pooled together using a random-effects model for assessing the EASI-75 EASI-75: Eczema Area and Severity Index-75

EASI-75: The EASI-75 was reported in all five included studies. It was observed that in comparison to the placebo arm, a higher number of patients achieved EASI-75 in the upadacitinib arm (R.R. = 3.86; 95% CI = 3.12 to 4.78, p < 0.00001). The test for heterogeneity was significant (χ2= 19.24, df=9 (p=0.02), I2 = 53%). The 30 mg group of upadacitinib demonstrated a better response than the 15 mg group in the AD patients as revealed by sub-group analysis (RR, 4.15 vs 3.63) (Figure 2)

**Figure 3:**
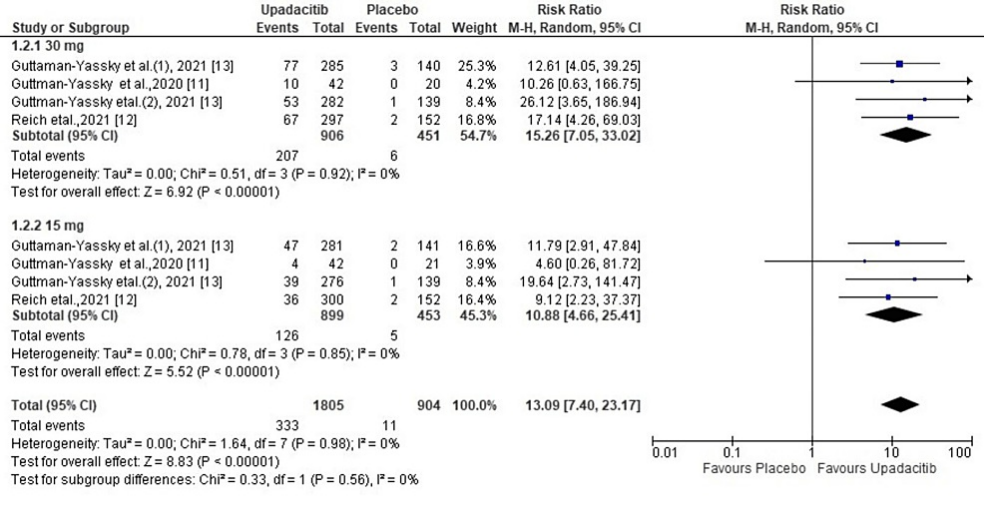
Forest plot of the included studies pooled together using a random-effects model for assessing the EASI-100 EASI-100: Eczema Area and Severity Index-100

ESAI-100: The EASI-100 at week 16 in patients receiving upadacitinib and patients receiving placebo was reported in four enrolled studies of our meta-analysis. As shown in Figure 3, the random effect model of pooled data reported that a significantly higher proportion of patients administered upadacitinib achieved EASI-100 as compared to those administered placebo (R.R. = 13.09; 95% CI = 7.40 to 23.17, p < 0.00001). However, the heterogeneity test was non-significant (χ2= 1.64, df= 7 (p=0.98), I^2^ = 0%). Sub-group analysis showed that 30 mg was associated with a better EASI-100 outcome than 15 mg. (RR, 15.26 vs 10.88) (Figure 3).

WP-NRS Response

**Figure 4:**
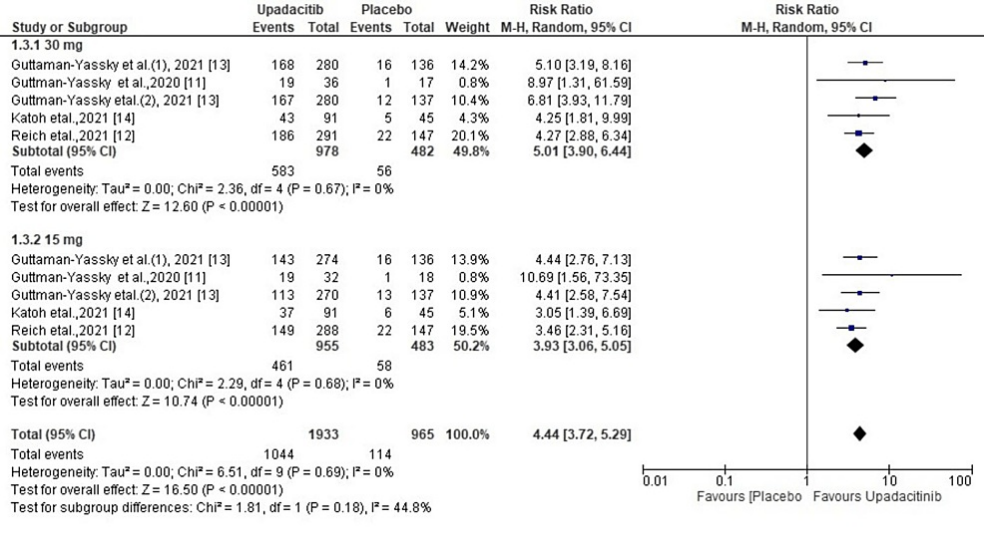
Forest plot of the included studies pooled together using a random-effects model for assessing the WP-NRS WP-NRS: Worst Pruritus Numerical Rating Score

All five studies included in our meta-analysis reported a WP-NRS response. The random effect model analysis showed that a significantly greater number of patients in the upadacitinib group achieved a WP-NRS response (R.R. = 4.44; 95% CI = 3.72 to 5.29, p< 0.00001) compared to the placebo. However, heterogeneity was not significant (χ2= 6.51, df=9 (p = 0.69), I^2^ = 0%). It was observed on sub-group analysis that the 30 mg dose demonstrated a better response than the 15 mg dose in AD patients with moderate to severe disease (RR, 5.01 vs 3.93) (Figure 4).

v-IGA AD Response

**Figure 5:**
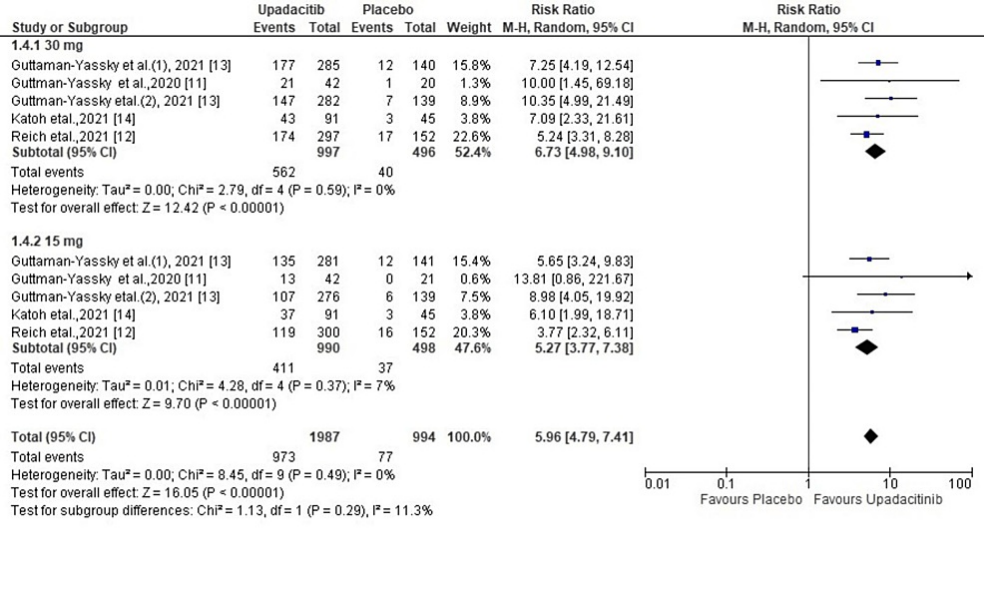
Forest plot of the included studies pooled together using a random-effects model for assessing v-IGA v-IGA: validated Investigator’s Global Assessment

All five included RCTs reported data regarding a v-IGA AD response between patients of the upadacitinib and placebo groups. Analysis of pooled data revealed that a significantly higher number of patients receiving upadacitinib achieved a v-IGA AD response at 16 weeks than those receiving a placebo (RR = 5.96; 95% CI = 4.79 to 7.41, p < 0. 00001), and heterogeneity was not significant (χ2= 18.13, df=9 (p=0.49), I^2^ = 0%). Subgroup analysis revealed that 30 mg had a better clinical response than 15 mg in AD patients with moderate to severe disease (RR, 6.73 vs 5.27) (Figure 5).

Treatment-Emergent Adverse Events

**Figure 6:**
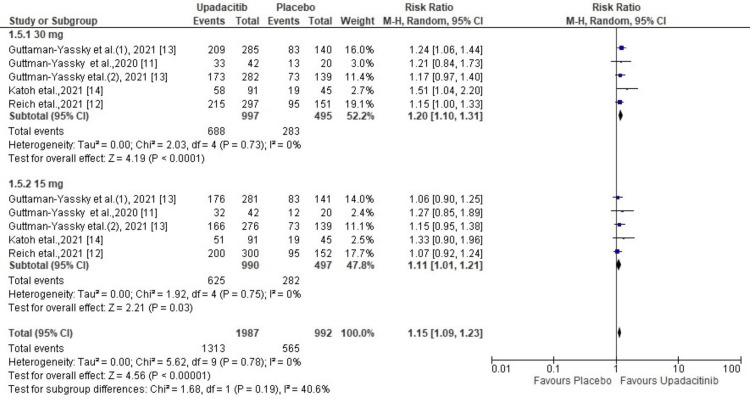
Forest plot of the included studies pooled together using a random-effects model for assessing the treatment-emergent adverse events

Treatment-emergent adverse events were reported in all five included studies of this meta-analysis. The risk of developing adverse events was comparatively higher in the upadacitinib treatment arm than in the placebo arm (R.R. = 1.15; 95% CI = 1.09 to 1.23, p<0.00001). The test for heterogeneity was not significant (χ2= 5.62, df=9 (p=0.78), I^2^ = 0%). Acne, upper respiratory tract infection, and nasopharyngitis were commonly reported adverse events. Subgroup analysis revealed that a 30 mg dose was associated with a higher proportion of TEAEs than those receiving a 15 mg dose (RR, 1.20 vs 1.11) (Figure 6).

Meta-Regression

No significant change in EASI 75, EASI 100, w-IGA, and WP-NRS response was observed upon meta-regression analysis when the dose of upadacitinib was increased from 15 mg to 30 mg (Table 3).

**Table 3:** Meta-regression comparing the effect of 15 and 30 mg doses of upadacitinib on study outcomes P< 0.05 is considered significant. EASI: Eczema Area and Severity Index; WP-NRS: Worst Pruritus Numerical Rating Score; v-IGA: validated Investigator’s Global Assessment; TEAE: treatment-emergent adverse events

SNo	Parameter	Beta estimate (Confidence interval)	P value
1.	EASI-75	0.0093(-0.0202 to 0.0387)	0.5372
2.	EASI-100	0.0226 (-0.0539 to 0.0990)	0.5632
3.	WP-NRS	0.0117(-0.0119 to 0.0353)	0.3327
4.	v-IGA	0.0172(-0.0119 to 0.0463)	0.2469
5.	TEAEs	0.0055(-0.0028 to 0.0137)	0.1943

Publication Bias

The publication bias for our meta-analysis was assessed using Egger’s regression test, which was found to be non-significant (p=0.0931).

Grade of Evidence

The Gradepro GDT tool used for assessing the certainty of the evidence showed that the overall certainty was ‘high’ for all the outcomes (Table 4).

**Table 4:** Certainty of evidence based on grade (upadacitinib vs. placebo in atopic dermatitis) EASI, eczema area and severity index; vIGA, validated investigator global assessment; WPNRS, worst pruritus numerical rating scale; TEAE, treatment-emergent adverse event

Certainty assessment							№ of patients		Effect		Certainty	
	№ of studies	Study design	Risk of bias	Inconsistency	Indirectness	Imprecision	Other considerations	Upadacitinib	Placebo	Relative (95% CI)		Absolute (95% CI)
EASI 75	5	randomized trials	not serious	not serious	not serious	not serious	none	1396/1987 (70.3%)	184/994 (18.5%)	RR 3.86 (3.12 to 4.78)	529 more per 1,000 (from 392 more to 700 more)	⨁⨁⨁⨁ High
EASI 100	4	randomized trials	not serious	not serious	not serious	not serious	none	333/1805 (18.4%)	11/904 (1.2%)	RR 13.93 (7.88 to 24.64)	157 more per 1,000 (from 84 more to 288 more)	⨁⨁⨁⨁ High
WPNRS	5	randomized trials	not serious	not serious	not serious	not serious	none	1044/1933 (54.0%)	114/965 (11.8%)	RR 4.44 (3.72 to 5.29)	406 more per 1,000 (from 321 more to 507 more)	⨁⨁⨁⨁ High
v-IGA	5	randomized trials	not serious	not serious	not serious	not serious	none	973/1987 (49.0%)	77/994 (7.7%)	RR 5.96 (4.79 to 7.41)	384 more per 1,000 (from 294 more to 497 more)	⨁⨁⨁⨁ High
TEAEs	5	randomized trials	not serious	not serious	not serious	not serious	none	1313/1987 (66.1%)	565/992 (57.0%)	RR 1.15 (1.09 to 1.23)	85 more per 1,000 (from 51 more to 131 more)	⨁⨁⨁⨁ High

Discussion

The therapeutic goals in AD are the amelioration of disease symptoms, prevention of exacerbations, and the overall well-being of affected patients [18]. In moderate to severe AD, topical corticosteroids and calcineurin inhibitors are considered the initial treatment options. However, the relapsing nature of the disease and the adverse-effect profile of corticosteroids necessitates the search for newer therapeutic agents that can achieve prolonged remission and will have a better long-term safety profile. Topical and oral formulations of JAK inhibitors are gradually emerging as a credible treatment option for AD patients [19]. Among the oral JAK inhibitors, abrocitinib, baricitinib, and upadacitinib have demonstrated promising results in a few recently conducted clinical trials [20-23].To our knowledge, this meta-analysis is the first to summarize the safety and efficacy of upadacitinib against a placebo in patients with AD having moderate-to-severe disease.

The EASI is a validated tool for grading the clinical signs of atopic dermatitis. Harmonising Outcome Measures in Eczema (HOME) has recommended EASI as a core instrument for evaluating the severity and extent of involvement in clinical trials of AD [24]. The disease characteristics of AD, such as eczema, induration, excoriation, and lichenification, are evaluated for severity in EASI [25]. EASI-75 is defined as more than or equal to a 75% improvement from the baseline EASI score, whereas EASI-100 indicates a 100% reduction from the baseline EASI score.

The pooled effect of 15 mg and 30 mg doses of upadacitinib on EASI-75 revealed a statistically significant response against placebo at week 16 (RR = 3.86, p < 0.00001). Similarly, the pooled effect of both doses of the drug showed a statistically significant response in EASI-100 at 16 weeks as compared to those administered placebo (RR = 13.93, p < 0.00001).

Validated Investigator Global Assessment for atopic dermatitis (vIGA AD) is a clinician-rated standardized tool for the assessment of disease severity in AD at a specific time point. Because of its reliability and validity, it is widely used as a primary endpoint measure for AD in clinical research [26]. We found that patients receiving 15 mg and 30 mg doses of upadacitinib had statistically significant vIGA responses compared to the placebo at week 16 (RR = 5.96, p < 0. 0001). WP-NRS was also one of the efficacy outcome measures in our study. WP-NRS is a reliable and sensitive, validated, self-reported scale to measure the intensity of itching in AD patients [27]. Our study demonstrated that a significant proportion of patients who received upadacitinib achieved the desired WP-NRS response at 16 weeks in comparison to patients who received a placebo (RR = 4.44, p< 0.00001).

A network meta-analysis conducted by Silverberg et al. also showed that patients in the upadacitinib 30 mg group had the highest efficacy in achieving EASI 75 [28]. Another network meta-analysis conducted by Wan et al. on the comparative efficacy and safety of abrocitinib, baricitinib, and upadacitinib in moderate to severe atopic dermatitis also revealed that upadacitinib 30 mg was superior in achieving EASI and IGA response in comparison to abrocitinib and baricitinib [29]. Zhang et al. have conducted a meta-analysis on the efficacy of JAK inhibitors upadacitinib and abrocitinib in patients with moderate to severe atopic dermatitis and found that upadacitinib had shown significant therapeutic response in EASI, IGA, and PNRS responders [30]. They have also concluded that upadacitinib had better efficacy than abrocitinib for EASI-75, IGA, and PNRS response. Although we have included a greater number of studies in our analysis, our overall observations regarding upadacitinib conform with these studies. In addition, we have also analyzed the effect of dose on these outcomes and found no significant association. Compared to other JAK1 inhibitors, upadacitinib exhibits a better pharmacological response possibly because of its additional effect in decreasing the production of pro-inflammatory mediators induced by IL-6, IL-15, IFN-α, and IFN-c [30].

Furthermore, it has been revealed in previous studies that upadacitinib has a relatively higher incidence of upper respiratory tract infection and nasopharyngitis, which is in consonance with our analysis. It was observed that patients in the upadacitinib treatment arm had an increased risk of TEAEs, including acne, upper respiratory tract infection, nasopharyngitis, and headache, and these adverse events have been reported as class effects. However, most of them were of the mild category and could be managed conservatively. Although the upadacitinib group had experienced more adverse effects compared to placebo, it did not lead to any disruption in treatment.

Though the certainty of the evidence of all the aforementioned outcome measures was "high," as suggested by the GRADEpro assessment, it should be cautiously interpreted as the studies included in this meta-analysis were only five in number.

Our study also has other limitations. The studies included in this meta-analysis had a modest number of participants and were of shorter duration. Further, in all studies, a placebo was the comparator rather than any active agent. Moreover, atopic dermatitis is a chronic disease affecting different age groups of patients with varying clinical features, thereby limiting the overall clinical inference that can be drawn from our meta-analysis. However, to the best of our knowledge, our study can be considered comprehensive, as it has included all the published clinical trials and has assessed the effect of 15 mg and 30 mg doses of upadacitinib on clinical outcomes.

## Conclusions

In conclusion, our study has provided preliminary proof that upadacitinib was an effective and safe targeted therapy for moderate to severe AD patients who do not respond to conventional treatment and need systemic management. However, studies with relatively larger sample sizes and longer durations of therapy comparing the efficacy and safety of upadacitinib head-to-head with active drugs will be essential to provide clear evidence about the use of the drug in this condition.
